# Cytotoxic and Antimicrobial Activity of the *Ageratina* Genus

**DOI:** 10.3390/molecules30234656

**Published:** 2025-12-04

**Authors:** Sarai Rojas-Jiménez, David Osvaldo Salinas-Sánchez, Verónica Rodríguez-López, Roberta Salinas-Marín, Dante Avilés-Montes, César Sotelo-Leyva, Rodolfo Figueroa-Brito, Genoveva Bustos Rivera-Bahena, Rodolfo Abarca-Vargas, Dulce María Arias-Ataide, María Guadalupe Valladares-Cisneros

**Affiliations:** 1 Laboratorio de Glicobiología Humana y Diagnóstico Molecular, Centro de Investigación en Dinámica Celular, Instituto de Investigación en Ciencias Básicas y Aplicadas, Universidad Autónoma del Estado de Morelos, Cuernavaca 62209, Morelos, Mexico; sarai.rojas@alumnos.uaem.mx (S.R.-J.); rsm@uaem.mx (R.S.-M.); 2 Facultad de Ciencias Químicas e Ingeniería, Universidad Autónoma del Estado de Morelos (UAEM), Av. Universidad 1001, Col. Chamilpa, Cuernavaca 62209, Morelos, Mexico; genobrb@uaem.mx; 3 Centro de Investigación en Biodiversidad y Conservación, Universidad Autónoma del Estado de Morelos (UAEM), Av. Universidad 1001, Col. Chamilpa, Cuernavaca 62209, Morelos, Mexico; 4 Escuela de Estudios Superiores Jicarero (EESJ), Universidad Autónoma del Estado de Morelos (UAEM), Carretera Galeana-Tequesquitengo s/n, Comunidad El Jicarero, Jojutla 62915, Morelos, Mexico; dulce@uaem.mx; 5 Facultad de Farmacia, Universidad Autónoma del Estado de Morelos (UAEM), Av. Universidad 1001, Col. Chamilpa, Cuernavaca 62209, Morelos, Mexico; veronica_rodriguez@uaem.mx; 6 Facultad de Ciencias Biológicas, Universidad Autónoma del Estado de Morelos (UAEM), Av. Universidad 1001, Col. Chamilpa, Cuernavaca 62209, Morelos, Mexico; dante.aviles@uaem.mx; 7 Facultad de Ciencias Químico-Biológicas (FCQB), Universidad Autónoma de Guerrero, Chilpancingo de los Bravo 39090, Guerrero, Mexico; cesarsotelo@uagro.mx; 8 Centro de Desarrollo de Productos Bióticos (CEPROBI-IPN), Yautepec 62730, Morelos, Mexico; rfigueroa@ipn.mx; 9 Facultad de Medicina, Universidad Autónoma del Estado de Morelos (UAEM), Leñeros, Esquina Iztazzíhuatl s/n, Col. Volcanes, Cuernavaca 62350, Morelos, Mexico; rodolfo.abarca@uaem.mx

**Keywords:** *Ageratina*, cytotoxicity, cancer cell lines, microorganisms, Gram-positive and Gram-negative microorganisms, yeast, fungus, protozoa, virus

## Abstract

Medicinal plants have long been used for therapeutic purposes in many cultures. They represent sources of important bioactive compounds, often of pharmacological significance. *Ageratina* Spach is the largest genus in Mexico and is characterised by its traditional use in the treatment of cancer and infections of the skin, blood, and intestines. Different species of *Ageratina* have been biologically evaluated at the extract and compound levels, and their chemical contents have been purified and characterised. Following a PRISMA meta-analysis, 29 scientific reports were selected and analysed. Tables of different *Ageratina* species were integrated to compare their cytotoxic and antimicrobial activity at the extract and compound levels. Twelve pure and isolated natural compounds were tested for cytotoxic activity against several cell lines from lung, colon, and breast cancer, cervical carcinoma, hepatocarcinoma, promyelocytic leukaemia, and histiocytic lymphoma. Forty-one pure and isolated natural compounds were evaluated for antimicrobial activity against a wide spectrum of microorganisms, including Gram-positive and Gram-negative bacteria, yeast, fungi, parasites and viruses. *Ageratina* Spach contains cytotoxic and antimicrobial substances with broad chemical profiles. In addition to being a plant with active compounds, it could be useful for future rational drug design.

## 1. Introduction

Cancer is a disease in which the cell cycle is deregulated by DNA damage, leading to uncontrolled cell growth and the potential for metastasis to different parts of the body. The most common treatment for cancer is chemotherapy, which produces side effects and can result in resistance to the compounds used [1]. Cancer is a prevalent disease worldwide. In 2022, there were already almost 20 million new cases globally, and by 2050, this number is projected to increase to approximately 33 million [2]. Global cancer incidence is a significant public health issue, and projections show a continued increase in cases and deaths, driven by factors like population growth and ageing [3,4]. On the other hand, antimicrobial-resistant infections caused by bacteria, fungi, parasites, and viruses are continually increasing worldwide, making it urgent to prevent and treat them effectively [5,6,7].

Worldwide research on medicinal plants is needed to develop new treatments for diseases. The World Health Organization (WHO) recognises the use of traditional medicine for therapeutic purposes, as it is the oldest known healthcare system, especially in developing countries [8]. Medicinal plants are an important source of natural products for treating different health problems. As research on medicinal plants has increased, about 80% of chemotherapeutic drugs have been derived from plants [9].

### Ethnobotany

**Figure 1:**
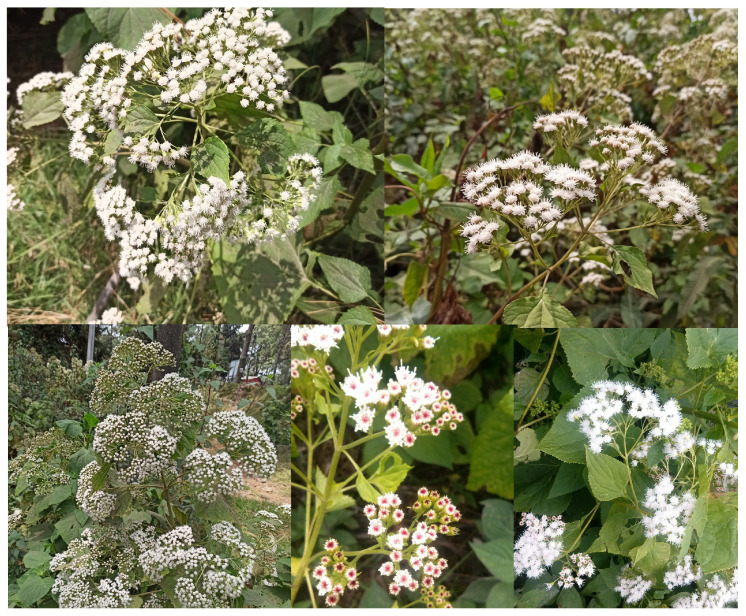
Aerial parts characteristics of *Ageratina* Spach.

*Ageratina* Spach is a native New World genus in the tribe Eupatorieae of the Asteraceae family [10]. This genus comprises 316 accepted species of *Ageratina*. They are distributed from the United States to South America; Mexico has the largest number of species. However, some species of *Ageratina* have been introduced into some countries in Europe, Africa, Asia, and Oceania [11]. *Ageratina* spp. are perennial herbs, shrubs, or small trees less than 10 m high. The florets are white or lavender, ranging from a few (about 10) to many per head (50 or more), with tubular corollas and poorly defined throats (Figure 1); apically, they include five lobes that are generally well defined, pubescent, or glabrous [12].

Species of the genus *Ageratina* grow in warm, humid climates with abundant rainfall, because these plants absorb water through their roots, retaining it in their stems, and benefiting from the humidity of their surroundings. Consequently, their distribution and dispersal are primarily in mountainous areas, near rivers, streams, or ravines [13]. Studies of some *Ageratina* species have demonstrated their ability to develop in a wide variety of soils with different pH levels, as well as tolerating some salinity and low nutrient levels. This has allowed species of this genus to be ecologically adaptable and widely distributed [14,15].

In Mexico, *Ageratina* spp. are widely used in traditional Mexican medicine, commonly known as ‘axihuitl’, which means water root. Axihuitl is very commonly used to treat numerous diseases, including persistent gastric ulcers, bacterial and fungal infections, and cancer. It is also employed to treat women after childbirth, fungal infections of the feet, and for heat treatment [16,17]. This review provides a current overview of scientific studies on *Ageratina* species, offering comprehensive and critical information on their chemistry and pharmacological activities worldwide from 2010 to 2025.

## 2. Results

**Figure 2:**
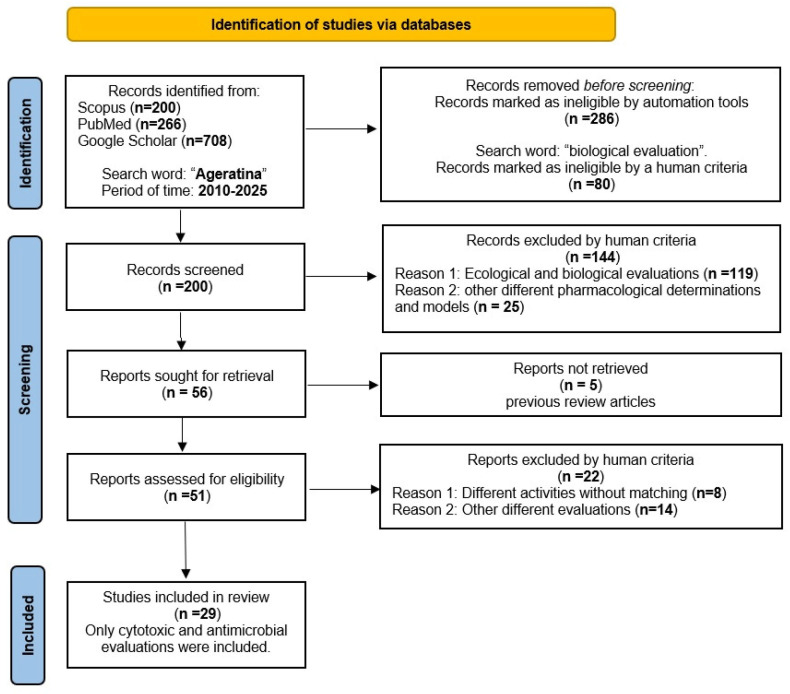
PRISMA meta-analysis flow diagram for systematic review of *Ageratina*.

The PRISMA guidelines [18] were used to ensure transparency and completeness of the systematic review process (Figure 2).

Identification: The researchers conducted a literature review across several databases from 2010 to 2025. The records identified 200 relevant studies on Scopus and 266 on PubMed. Additionally, a manual search using Google Scholar found 708 studies. In addition to the automated database search to ensure that no relevant studies were overlooked (286), 80 studies were removed using the term ‘biological evaluation’; they were marked as ineligible. Screening: After removing duplicates, 200 articles remained. After reviewing the titles and abstracts, 144 articles were excluded, leaving 56 for full-text screening. Eligibility: The remaining articles were read in full and assessed. Five studies were excluded as they were reviews. Then, 51 reports were assessed, and 22 were excluded because they included different activities, and some others were from different evaluations.

Inclusion: Finally, 29 papers were included in this systematic review, all of which reported cytotoxic and antimicrobial activities. These articles were analysed in detail to answer the study’s research question.

Table 1 shows the different species of the *Ageratina* genus identified by the PRISMA meta-analysis and included in this review. The species are presented in alphabetical order.

Table 2 shows the cytotoxic activity of the organic and aqueous extracts obtained from different *Ageratina* species.

*Ageratina gracilis* is a unique species that has lower IC_50_ values at the extract level; the inflorescent petrol extract was active against HT29 cells (IC_50_ = 12.67 + 1.13 μg/mL) and the leaf petrol extract was active against HT29 (IC_50_ = 11.20 + 1.20 μg/mL); SiHa (IC_50_ = 12.91 + 0.92 μg/mL), and MDA-MB-231 cells (IC_50_ = 14.72 + 0.69 μg/mL) [36].

Table 3 shows the cytotoxic activity of the diverse pure isolated natural compounds from the different *Ageratina* species.

**Figure 3:**
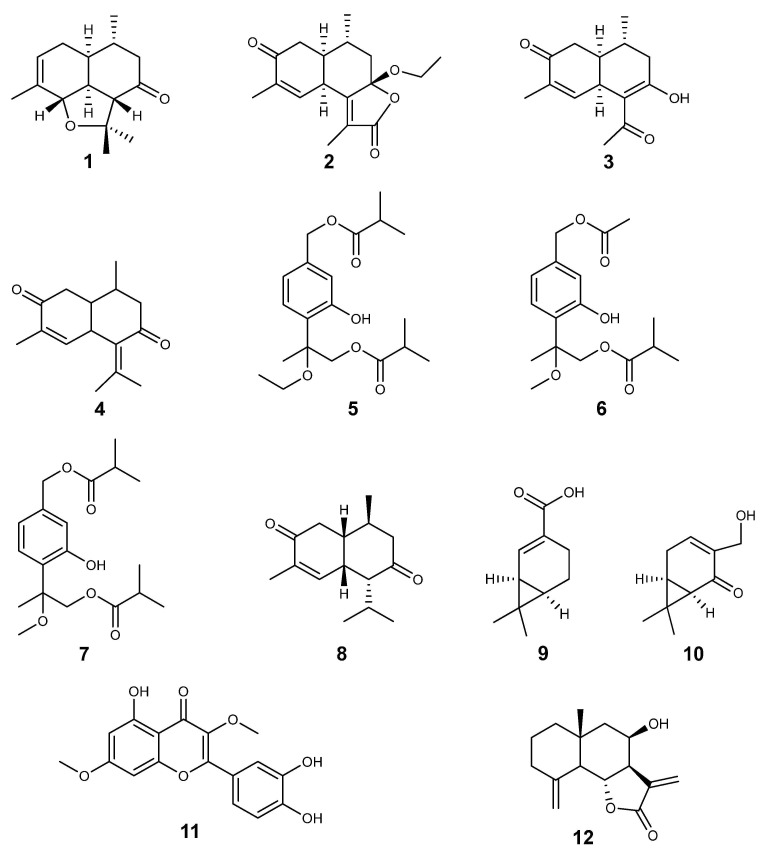
Structure of compounds isolated from *Ageratina* Spach.

The pure natural compounds isolated from *Ageratina* Spach and bio-evaluated in an *in vitro* anticancer assay are shown in Figure 3.

According to the IC_50_ values established to distinguish an active cytotoxic substance [5], the compounds with good cytotoxic activity were from *Ageratina adenophora*. A new tricyclic cadinene (**1**) was isolated and moderately active against A549 (IC_50_ = 11.45 + 0.69 μM) and SMMC-7721 cells (IC_50_ = 9.96 + 1.45 μM). Also, (+)-(5*R*,7*S*,9*R*,10*S*)-2-oxocadinan-3,6(11)-dien-12,7-olide (**2**) was isolated, which was moderately active against A549 (IC_50_ = 9.85 + 0.88 μM), SMMC-7721 (IC_50_ = 13.44 + 2.32 μM), and MDA-MB-231 cells (IC_50_ = 12.72 + 1.58 μM). Cadinene norsesquiterpenoid (**3**) was isolated from *A. adenophora* and was moderately active against SMMC-7721 cells (IC_50_ = 10.28 + 1.67 μM) [27]. 9-oxo-10,11-dehydro-ageraphorone (Euptox A) (**4**) was cytotoxically active against HeLa (IC_50_ = 0.55 + 0.05 mg/mL), Caco-2 (IC_50_ = 1.43 + 0.08 mg/mL), and MCF-7 cells (IC_50_ = 1.63 + 0.08 mg/mL) [23]. Quercetin 3,7-dimethylether (11), isolated from *Ageratina dictyoneura*, was strongly active against U-937 cells (IC_50_ = 7.0 + 0.5 μM), and 8β-hydroxy-β-cyclocostunolide (**12**), isolated from *Ageratina illita*, was also strongly active against U-937 cells (IC_50_ = 6.0 + 0.7 μM) [34].

Table 4 shows the antimicrobial activity of the organic and aqueous extracts from *Ageratina* evaluated *in vitro* against different microorganisms: Gram-positive and Gram-negative cocci and bacilli, and yeast, fungi, protozoa, and viruses.

According to the minimal inhibitory concentration (MIC) values for distinguishing an active antimicrobial substance [48], we observed that the essential oil from *Ageratina pentlandiana* leaves was active against *S. aureus* (MIC = 11.9 + 0.1 μL/mL), *Bacillus subtilis* (22.7 + 0.3 μL/mL), *Escherichia coli* (57.7 + 0.1 μL/mL), and *Salmonella thyphimurium* (41.6 + 0.1 μL/mL) [45].

Table 5 shows the antimicrobial activity of the compounds isolated from species of *Ageratina* Spach and evaluated *in vitro* against different microorganisms: Gram-positive and Gram-negative cocci and bacilli, yeast, fungi, protozoa, and viruses.

**Figure 4:**
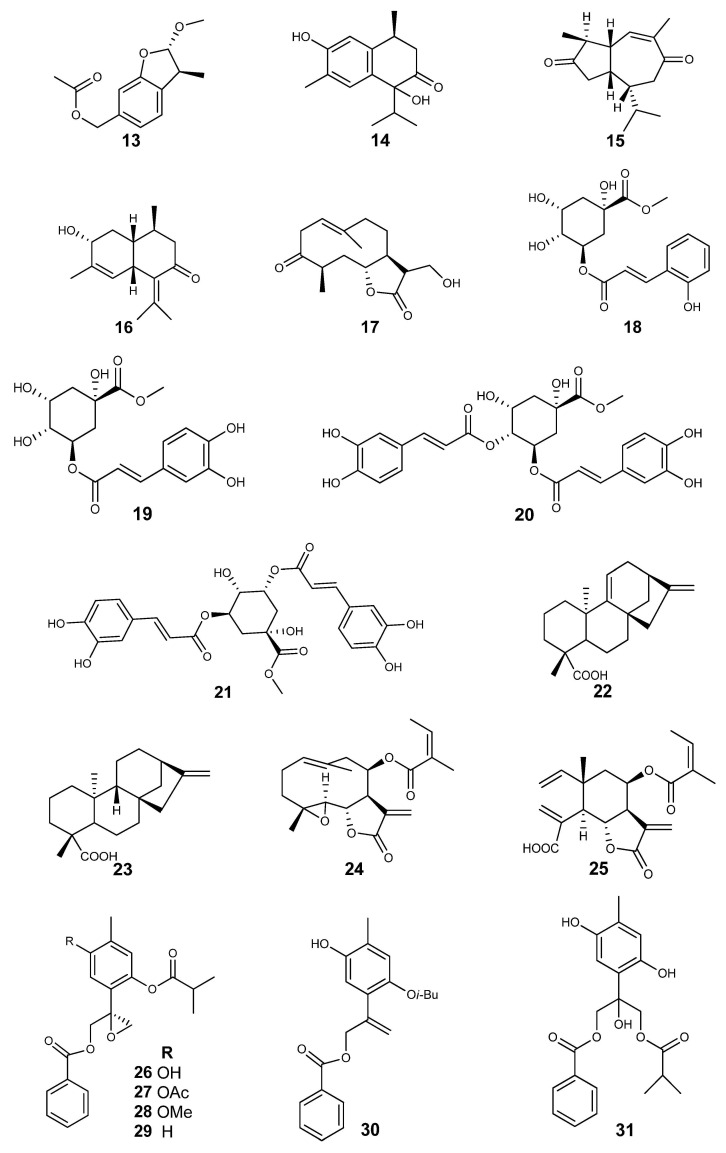

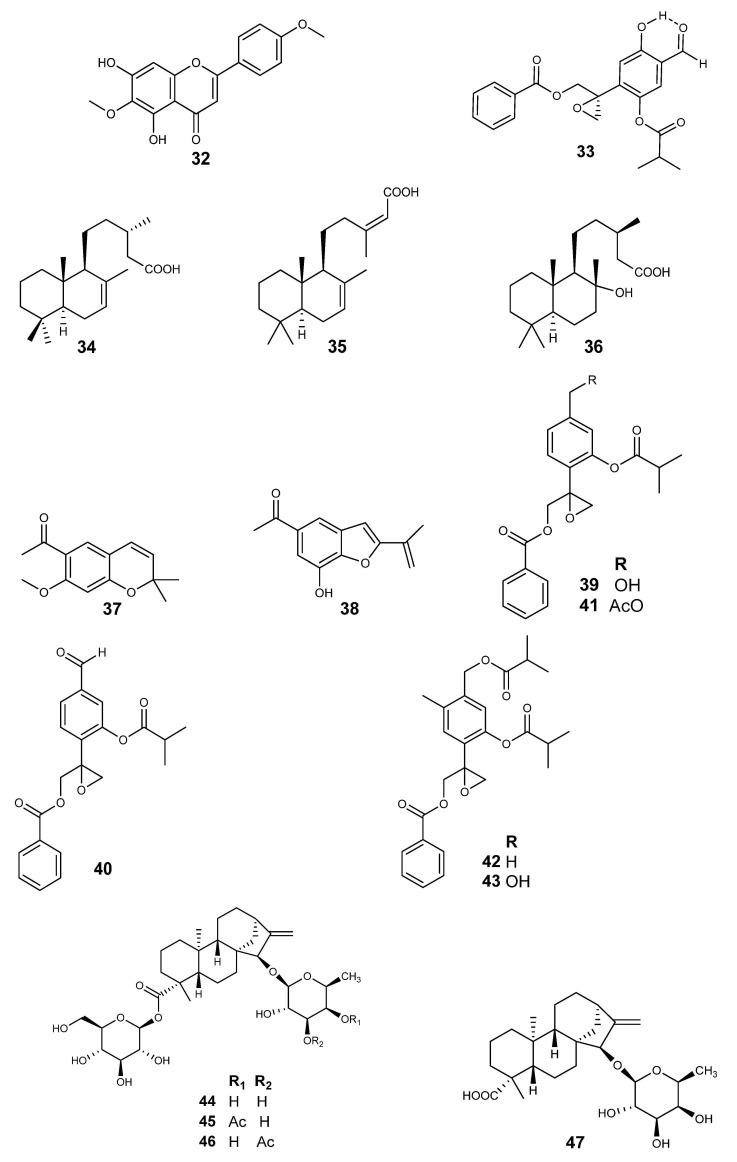
Structure of compounds isolated from *Ageratina* Spach evaluated as antimicrobials.

The pure natural compounds isolated from *Ageratina* Spach and bio-evaluated *in vitro* antimicrobial assays are shown in Figure 4.

Deltoidin A (**24**) from *Ageratina deltoide* was active against *E. coli* (MIC = 16 μg/mL) [32]. (8*S*)-10-Benzoyloxy-8,9-epoxy-6-hydroxythymol isobutyrate (**26**) was active against *Entamoeba histolytica* (IC_50_ = 1.6 μM). 10-Benzoyloxy-8,9-epoxy-6-acetyloxythymol isobutyrate (**27**) was active against *E. histolytica* (IC_50_ = 0.84 μM) and *Giardia lamblia* (IC_50_ = 24.4 μM) [35]. (−)-(5*S*,9*S*,10*S*,13*S*)-labd-7-en-15-oic acid (**34**) was active against *Staphylococcus aureus* (MIC = 0.78 mg/mL) and *Bacillus subtilis* (MIC = 0.15 mg/mL); (−)-(5*S*,9*S*,10*S*,13*Z*)-labda-7,13-dien-15-oic acid (**35**) was active against *S. aureus* (MIC = 1.0 mg/mL). (+)-(5*S*,8*R*,9*R*,10*S*,13*R*)-8-hydroxylabdan-15-oic acid (**36**) was active for *S. aureus*; all of these compounds were isolated from *Ageratina jocotepecana* [41]. Encecalin (**37**) was active against *Fusarium oxysporum*, producing a 10 mm inhibition zone. 7-hydroxy-dehydrotremetone (**38**) produced an inhibition zone for *Colletotrichum gloesporoides* (17.28 mm), *Colletotrichum musae* (17.24 mm), *Rhizoctonia solani* (16.40 mm), *F. oxysporum* (13.90 mm), and *Alternaria alternata* (14.36 mm). All of these compounds were isolated from *Ageratina adenophora* [25]. All compounds isolated from *Ageratina cylindrica* were active for *E. histolytica* and *G. lamblia* [30].

## 3. Discussion

The study of medicinal plants through their organic or aqueous extracts and their pure natural products continues to be of great importance, because they afford a wide range of compounds with pharmacological activity against significant diseases, such as cancer and microbial, fungal, parasitic, and viral infections, among others. According to the WHO, these diseases are the leading causes of mortality worldwide [5,48]. For this purpose, it is of the utmost importance to conduct reviews of scientific studies that provide an overview of the extracts and molecules isolated from plants used in traditional medicine, such as those of the *Ageratina* Spach. In this review, 29 scientific papers were selected through a PRISMA meta-analysis from 2010 to 2025, covering the anticancer and antimicrobial effects of 47 compounds isolated from 16 species of the *Ageratina* genus.

The criteria for cytotoxic activity of a plant as a promising crude extract for the purification of active compounds, according to the American National Cancer Institute (NCI), USA, are an IC_50_ < 20 μg/mL [48,49,50,51] and an IC_50_ < 4 μg/mL (or <10 μM) for an moderately and high active cytotoxic natural pure compound, respectively [52]. The cytotoxic activity at extract level of the inflorescent petrol extract from *A. gracilis* had lower IC_50_ values against SiHa (IC_50_ = 21.19 ± 1.25 μg/mL) and HT29 cells (IC_50_ = 12.67 ± 1.13 μg/mL) and the petrol extract of the leaves from the same species was active against SiHa (IC_50_ = 12.91 ± 0.92 μg/mL), HT29 (IC_50_ = 11.20 ± 1.20 μg/mL), and MDA-MB-231 cells (IC_50_ = 14.72 ± 0.69 μg/mL) [36]. Those indicate that nonpolar extract from *A. gracilis* contains interesting cytotoxic active compounds.

A variability of active pure compounds isolated from *Ageratina* Spach, according to their chemical structure, were terpenes (mono- and sesqui-), cinnamic acids, flavonoids, and their glucosides. Twelve pure natural compounds isolated from *Ageratina* species were evaluated against different human cancer cell lines, including breast, lung, hepatic, uterine, colon, skin, prostatic, leukaemia, and lymphoma. The results showed that, according to the IC_50_ values established from NCI, the active cytotoxic pure natural products were a new tricyclic cadinene (**1**) isolated from *A. Adenophora*, which was active against A549 (IC_50_ = 11.45 ± 0.69 μM) and SMMC-7721 cells (IC_50_ = 9.96 ± 1.45 μM). The (+)-(5*R*,7*S*,9*R*,10*S*)-2-oxocadinan-3,6(11)-dien-12,7-olide (**2**) was also isolated from *A. Adenophora,* and was active against A549 (IC_50_ = 9.85 ± 0.88 μM), and MDA-MB-231 cells (IC_50_ = 12.72 ± 1.58 μM). Cadinene norsesquiterpenoid (**3**) was also isolated from *A. Adenophora* and was active only against SMMC-7721 cells (IC_50_ = 10.28 ± 1.67 μM) [27]. On the other hand, Quercetin 3,7-dimethylether (**11**), isolated from *A. dictyoneura*, was active against U-937 cells (IC_50_ = 7.0 ± 0.5 μM) and 8β-hydroxy-β-cyclocostunolide (**12**), isolated from *A. illita*, was also active against U-937 cells (IC_50_ = 6.0 ± 0.7 μM) [34].

Compounds **1**, **2**, and **3** are sesquiterpene lactones isolated from *A. adenophora*. Compounds **1** and **2** were reported to be active for A549 cells. Compounds **1** and **3** were reported to be active for human hepatocellular carcinoma (SMMC-7721), and compound **2** was active for breast cancer (MDA-MB-231). Likewise, compound **12** isolated from *A. dictyoneura* is also a lactone, and it was active against histiocytic lymphoma (U937 cells) like the flavonoid **11** isolated from *A. illita*. Lactones consistently induce programmed cell death in lung, breast, liver, and leukaemia cancer cell lines [53,54,55]. And flavonoids induce apoptosis, affecting the cell cycle and modulating crucial signalling pathways [55,56]. The molecular mechanisms for cytotoxic activity from lactones and flavonoids are associated with alterations in the balance of Bcl-2 family proteins (increasing pro-apoptotic Bax and decreasing anti-apoptotic Bcl-2) and the activation of caspases-9 and -3 [57,58,59,60]. Other observations are that lactones target crucial signalling cascades, vital for cancer cell survival and progression. A key mechanism involves suppressing the NK-kB pathway, which is often overactive in cancer cells and regulates genes for survival, proliferation, and inflammation [61,62,63].

Likewise, plants contain abundant compounds, some of which have antimicrobial properties, providing them with protection against aggressors, especially microorganisms. Since the earliest times, plants have been used by various communities as medicine to treat a wide range of diseases, including infections. Multiple studies on medicinal plants have been conducted because they constitute a potential source of interesting compounds that warrant investigation for their antimicrobial activity and possess chemical structures that support further study and design to enhance the effects of new medicines [64]. The criteria for selecting organic or aqueous plant extracts and pure natural compounds with potential antimicrobial activity are based on the MIC, a key criterion used by many scientists to select antimicrobial botanicals. Plant extracts with MICs ≤ 100 μg/mL reveal significant antimicrobial activity. However, pure natural compounds with MICs < 10 μg/mL (or <25 μM) indicate strong antimicrobial activity, a characteristic of many pure natural compounds, and suggest that they may be considered potential candidates for developing a novel low-toxicity antitumor agent [65,66,67].

According to the MIC values for distinguishing active antimicrobial substances [64,68], the antimicrobial activities of *Ageratina* species against fungi, yeast, protozoa, and viruses have been observed in a few pure natural compounds. Macranthoin F (**20**) and Macranthoin G (**21**) were active against *S. enterica*, with MICs of 14.7 and 7.4 μM, respectively; those compounds were isolated from *A. adenophora* [24]. On the other hand, (8S)-10-Benzoyloxy-8,9-epoxy-6-hydroxythymol isobutyrate (**26**) and 10-Benzoyloxy-8,9-epoxy-6-acetyloxythymol isobutyrate (**27**) isolated from *Ageratina glabrata* were bioactive against *E. histolytica*, IC_50_ = 1.6 and 0.84 μM, respectively [35]. These important findings allow us to observe the activity of these natural compounds against opportunistic enteric pathogens, many of which are now multi-resistant [69,70].

Quinic acid derivatives such as caffeoylquinic acid ester and compounds **20** and **21** employ multi-targeted mechanisms against *S. enterica*, primarily by interfering with bacterial virulence, disrupting cell membrane integrity, and modulating host immune responses [71,72]. Quinic acid and its derivatives increase the permeability of the microorganism membrane and disrupt the cell wall structure, leading to leakage of intracellular components and cell death [73].

The high lipophilicity of monoterpenes (such as thymol and carvacrol) and their derivatives, like compounds **26** and **27**, is a multi-targeted approach that disrupts membrane integrity against Entamoeba histolytica, which is the primary mechanism of action. They readily integrate into the lipid bilayer of the parasite’s membrane and increase permeability, causing microorganisms to die by depolarization and a critical ionic disruption in homeostasis and enzymatic functions, which are necessary for survival. The compounds interact with and may cause oxidation of vital parasite proteins, including signalling molecules and oxidoreductases, thereby triggering parasite death [74,75,76].

The results indicate that natural compounds isolated from *Ageratina* Speech exhibit low activity against microorganisms associated with important infections such as amoebiasis, as well as giardiasis, which cause chronic diarrhoea [77,78,79]. However, it is important to continue to carry out investigations into medicinal plants used in traditional medicine against methicillin-resistant *S. aureus* and fungi, parasites, and viruses, because they contain valuable compounds that could exhibit effective activity against currently resistant pathogens [80,81,82].

## 4. Materials and Methods

A search for scientific studies on different species of *Ageratina* Spach was conducted across the Springer^®^, PubMed^®^, ScienceDirect^®^, and Google Scholar^®^ online databases. The searching methodology used in this work is as follows:

*Information sources*: The search is conducted across multiple electronic databases, including Scopus, PubMed, and Google Scholar.

*Search terms*: A detailed search string will be constructed using a combination of keywords, Boolean operators, and medical subject headings (MeSH). For example: (‘cytotoxic activity’ AND ‘antimicrobial activity’ AND ‘*Ageratina*’), (‘anticancer activity’ OR ‘antifungal’ OR ‘antiviral’ OR ‘*Ageratina*’).

*Search limitations*: Searches are limited by date to the timeframe 2010 to 2025.

*Additional sources*: The reference lists of included studies and relevant review articles will be hand-searched to find additional studies.

*Population/Intervention*: Studies that investigate the cytotoxic or antimicrobial activity of specific *Ageratina* species.

*Outcome*: Quantitative data on cytotoxic effects (e.g., IC_50_ values) and antimicrobial effects (e.g., minimum inhibitory concentration—MIC, or zone of inhibition).

*Study design*: Eligible study designs typically include *in vitro* and, if relevant, *in vivo* studies, depending on the review’s scope.

*Selection process*: The screening process will involve two independent reviewers to reduce bias.

*Title and abstract screening*: Reviewers will screen titles and abstracts based on the eligibility criteria.

*Full-text review*: The full text of potentially eligible studies will be retrieved and assessed.

*Documentation*: The study selection process will be summarised in a PRISMA 2020 flow diagram, clearly indicating the number of studies identified, screened, and ultimately included or excluded.

*Data extraction form*: A standardised, pre-designed data extraction form will be used to ensure consistency.

*Study characteristics*: Author, year of publication, study design, and location.

*Compound details*: Name, source (plant, plant extract, isolated compound), concentration tested, and other relevant properties.

*Cytotoxic data*: Cell line tested, duration of exposure, and quantitative measures such as IC_50_ values.

*Antimicrobial data*: Pathogen tested (e.g., bacterium, fungus), assay method, and quantitative measures such as MIC or zone of inhibition.

*Cytotoxicity*: Quantitative pooling of IC_50_ values may be possible for studies using the same cell line and compound class.

*Antimicrobial activity*: Pooled estimates for MIC values or zones of inhibition may be calculated for studies focusing on the same pathogen and compound.

## 5. Future Perspectives

A few of the globally medicinal plants have been scientifically investigated. An infinite number of bioactive compounds, chemical and pharmacological, are unexplored and underexploited in medicinal plants. Traditional Latin America’s medicine, particularly traditional Mexican medicine [83], is an important natural source for the discovery of cytotoxic and resistance compounds that could become useful therapeutic tools [84,85,86].

However, the interactions between medicinal plant extracts and anticancer or antimicrobial agents can be either favourable, such as synergism, or harmful, as in antagonism [87]. Therefore, further studies are required, especially *in vivo* studies and research on the toxicity of these products, for them to be recognised as biomedical agents.

This work shows the considerable potential of complex natural compound mixtures from the *Ageratina* species, and suggests future investigations involving precise chemical and scientific explorations, because this species has adequate *in vitro* cytotoxic and antimicrobial potential. In the future, this should be followed by *in vitro* testing with other cancer cells or *in vivo* testing in infected animal models to determine the preclinical relevance of such compounds and to establish a valid correlation with the *in vitro* efficacy results [88]. New studies should include molecular docking, structural chemical modifications, or synthesis of derivatives to improve pharmacokinetics and pharmacodynamics, as well as chemical structure–activity relationship analysis or green synthesis of nanoparticles [81,89].

Advanced techniques of biotechnological, genomics, proteomics, and metabolomics are nowadays applied to the research of medicinal plants and contribute to the advancement of alternative natural antimicrobials, characterisation of their interactions, and elucidation of the mechanisms of natural compounds’ action [90,91,92].

Likewise, further studies are required for extracts and individual isolated natural compounds. We especially suggest conducting toxicity assays and toxic *in vivo* studies of these natural compounds in order for them to be recognised as biomedical agents [93].

## 6. Conclusions

In summary, this work compiles the studies on *Ageratina* Spach published from 2010 to 2025; these medicinal plants are a natural source of compounds with potent antimicrobial activity and moderate cytotoxicity. Sixteen species of *Ageratina* have been studied; however, *A. adenophora* is the predominant species and has received more research attention. Additionally, 47 compounds included in this review showed chemical structures such as terpenes (mono- and sesqui-), cinnamic acids, flavonoids, and their glucosides, all of which are natural products that exhibit antimicrobial activity.

Medicinal plants have been extensively studied as a potential source of compounds used in the treatment of various human diseases, including cancer, antibiotic-resistant microorganisms, parasites, and viruses. Public health is becoming a global issue; it is crucial to evaluate the potential of plant-derived natural products and assess how well they can combat both current and emerging pathogens.

## Figures and Tables

**Table 1:** Species of *Ageratina* analysed in the review, from 2010 to 2025.

Species	Collection Place	Part of the Plant	Extract Solvent	Refs.
*A. adenophora*	Thailand	L	Ethanol	[19]
	Nepal	AP	Methanol	[20]
	India	L	Hydroalcoholic	[21]
	India	L	Methanol	[22]
	Portugal	L	Aqueous	[23]
	China	AP	Ethanol	[24]
	China	R	Methanol	[25]
	China	R	Ethanol	[26,27,28]
*A. cylindrica*	México	L	DCM	[29]
	México	L	Aqueous	[30]
	México	L	Petrol	[31]
*A. deltoidea*	Mexico	AP	Hexane	[32]
*A.* *dendroides*	Ecuador	L&F	EO-Hdest	[33]
*A. dictyoneura*	Dominican Republic	AP	Ethanol	[34]
*A. glabrata*	Mexico	L	DCM	[35]
*A.* *gracilis*	Colombia	I	Ethanol	[36]
	Colombia	I	Petrol	
	Colombia	L	Ethanol	
	Colombia	L	Petrol	
*A.* *havanensis*	Cuba	L	Ethanol	[37]
	Cuba	L	EtOAc	
	Cuba	L	n-butanol	
	Cuba	S	Ethanol	
	Cuba	S	EtOAc	
	Cuba	S	n-butanol	
	Cuba	S	Ethanol	
	Cuba	L&F	EtOAc	[38]
	Cuba	L&F	n-butanol	
	Cuba	L (FS)	Ethanol	[39]
	Cuba	L (FS)	EtOAc	
	Cuba	L (FS)	n-butanol	
	Cuba	S (FS)	Ethanol	
	Cuba	S (FS)	EtOAc	
	Cuba	S (FS)	n-butanol	
	Cuba	F (FS)	Ethanol	
	Cuba	F (FS)	EtOAc	
	Cuba	F (FS)	n-butanol	
*A. illita*	Dominican Republic	AP	Ethanol	[34]
*A.* *janni*	Venezuela	L	EO-Hdest	[40]
*A. jocotepecana*	Mexico	F	Hexane	[41]
	Mexico	L		
*A.* *popayanensis*	Colombia	AP	Hydroalcoholic	[42]
*A.* *pichinchensis*	Mexico	AP	(7:3) H-EtOAc	[43]
	Mexico	AP	Aqueous	[44]
	Mexico	AP	(7:3) H-EtOAc	
	Venezuela	L	EO-Hdest	[40]
*A.* *pentlandiana*	Peru	L	EO-Hdest	[45]
*A. tinifolia*	Colombia	AP	EO-Hdest	[46]
*A.* *vacciniaefolia*	Colombia	L	Ethanol	[47]

L = leaves, AP = Aerial parts; R = Root(s), S = Steams; F = Flowers; I = inflorescence; L&F = Leaves and flowers, FS = Flowering stage, DCM = Dichloromethane, H-EtOAc = Hexane-Ethyl acetate, EtOAc = Ethyl acetate, EO-Hdest = Essential oil by Hydro distillation.

**Table 2:** The cytotoxic activity of the extracts obtained from *Ageratina* Spach.

Specie	PP/ES	CCL	Result IC_50_ (μg/mL)	Ref.
*A. adenophora*	L/Hydroalcoholic	HCT-116	65.65 ± 2.1	[21]
	L/Methanol	A549	50.08 ± 0.14	[22]
	L/Aqueous	HeLa	950 ± 0.07	[23]
		Caco-2	289 ± 0.12	
		MCF-7	302 ± 0.16	
*A. havanensis* (FS)	L/Ethanol	4T1	381.6 ± 7.5	[39]
	L/EtOAc		252.5 ± 10.1	
	L/n-butanol		302.0 ± 8.0	
	S/Ethanol		228.2 ± 8.7	
	F/Ethanol		263.5 ± 8.2	
	F/EtOAc		259.5 ± 10.6	
	F/n-butanol		315.5 ± 9.9	
*A. havanensis* (VSt)	L/Ethanol	4T1	392.8 ± 6.7	
	L/EtOAc		313.0 ± 12.1	
	L/n-butanol		496.5 ± 6.7	
	S/Ethanol		355.7 ± 7.6	
*A. gracilis*	I/Ethanol	SiHa	41.14 ± 1.02	[36]
		HT29	62.33 ± 1.24	
		A549	74.56 ± 0.95	
		MDA-MB-231	62.81 ± 0.37	
		PC-3	77.35 ± 1.06	
	I/Petrol	SiHa	21.19 ± 1.25	
		HT29	12.67 ± 1.13	
		A549	38.50 ± 1.18	
		MDA-MB-231	26.94 ± 1.05	
		PC-3	52.65 ± 0.64	
	L/Ethanol	SiHa	71.98 ± 1.53	
		HT29	53.05 ± 0.82	
		A549	116.96 ± 1.04	
		MDA-MB-231	58.44 ± 0.78	
		PC-3	72.46 ± 0.47	
	L/Petrol	SiHa	12.91 ± 0.92	
		HT29	11.20 ± 1.20	
		A549	34.80 ± 1.15	
		MDA-MB-231	14.72 ± 0.69	
		PC-3	29.85 ± 1.27	
*A. pichinchensis*	L&S/Aqueous	KB	≥20	[44]
		HCT-15	≥20	
		UISO	≥20	
		OVCAR	≥20	
	L&S/(7:3) H-EtOAc	KB	≥20	
		HCT-15	≥20	
		UISO	≥20	
		OVCAR	≥20	
*A. popayanensis*	AP/Hydroalcoholic	Calu-1	444	[42]
		HepG2	387	
*A. havanensis*	L/Ethanol	Vero	CC_50_ (µg/mL) 2834 ± 448	[37]
	L/EtOAc	Vero	1670 ± 0.2	
	L/n-butanol	Vero	404.9 ± 43.5	
	S/Ethanol	Vero	5685 ± 117	
	S/EtOAc	Vero	2270 ± 99.9	
	S/n-butanol	Vero	457.4 ± 28.1	
	L&F/EtOAc	Vero	1242.8 ± 37.2	[38]

PP/ES = Part of the plant/extraction solvent, L = leaves, AP = Aerial parts; S = Steams; F = Flowers; L&F = Leaves and flowers; I = inflorescence; L&S = Leaves and Steams, FS = Flowering stage, VSt = Vegetative stage, H-EtOAc = Hexane-Ethyl acetate, EtOAc = Ethyl acetate, CCL = Cancer cell line; IC_50_: half-maximal Inhibitory Concentration; CC_50_: 50% Cytotoxic Concentration; A549: lung cancer; Caco-2: Colon cancer; Calu-1: epidermoid carcinoma of the lung; HCT-116: Adherent cell of colon cancer; HCT-15: colorectal carcinomas; HeLa: cervical carcinoma; HepG2: Hepatocarcinoma; HT29: colorectal adenocarcinoma; KB: Epithelial carcinoma; MCF-7: Breast cancer; MDA-MB-231: Human breast cancer; OVCAR: Ovarian adenocarcinoma; PC-3: Prostatic adenocarcinoma; SiHa: Uterine squamous cell carcinoma; 4T1: breast cancer; UISO: Merkel carcinoma cell; Vero: kidney-derived epithelial cell.

**Table 3:** The cytotoxic activity of the compounds obtained from *Ageratina* Spach.

Specie	Compounds	BioMod	CCL	Result IC_50_ μM	Ref.
*A. adenophora*	New tricyclic cadinene (**1**)	MTS	HL-60	24.06 ± 2.21	[26]
			A549	11.45 ± 0.69	
			SMMC-7721	9.96 ± 1.45	
			MDA-MB-231	16.35 ± 3.32	
			SW480	28.75 ± 3.93	
	(+)-(5*R*,7*S*,9*R*,10*S*)-2-oxocadinan-3,6(11)-dien-12,7-olide (**2**)		HL-60	35.73 ± 1.51	
			A549	9.85 ± 0.88	
			SMMC-7721	13.44 ± 2.32	
			MDA-MB-231	12.72 ± 1.58	
			SW480	26.03 ± 2.91	
	Cadinene norsesquiterpenoid (**3**)		HL-60	42.85 ± 1.35	
			A549	21.82 ± 0.65	
			SMMC-7721	10.28 ± 1.67	
			MDA-MB-231	30.42 ± 2.24	
			SW480	23.65 ± 1.49	
	9-oxo-10,11-dehydro-ageraphorone (Euptox A) (**4**)		HeLa	(mg/mL) 0.55 ± 0.05	[23]
			Caco-2	1.43 ± 0.08	
			MCF-7	1.63 ± 0.08	
	7,9-diisobutyryloxy-8-ethoxythymol (**5**)	MTT	A549	IC_50_ (μM) >100	[27]
			HeLa	>100	
			HepG2	>100	
	7-acetoxy-8-methoxy-9-isobutyryloxythymol (**6**)		A549	>100	
			HeLa	>100	
			HepG2	>100	
	7,9-diisobutyryloxy-8-methoxythymol (**7**)		A549	>100	
			HeLa	>100	
			HepG2	>100	
	9-oxoageraphorone (**8**)		A549	>100	
			HeLa	>100	
			HepG2	>100	
	(−)-isochaminic acid (**9**)		A549	32.37 ± 3.75	
			HeLa	25.64 ± 2.34	
			HepG2	41.87 ± 6.53	
	(1α,6α)-10-hydroxy-3-carene-2-one (**10**)		A549	30.65 ± 3.87	
			HeLa	18.36 ± 1.72	
			HepG2	39.44 ± 3.61	
*A. dictyoneura*	Quercetin 3,7-dimethylether (**11**)		U-937	7.0 ± 0.5	[34]
*A. illita*	(8*R*)-8-hydroxy-β-cyclocostunolide (**12**)			6.0 ± 0.7	

BioMod = Biological model, CCL = Cancer cell line, MTT: 3-[4,5-dimethylthiazol-2-yl]-2,5 diphenyl tetrazolium bromide; MTS: 3-(4,5-dimethylthiazol-2-yl)-5-(3-carboxymethoxyphenyl)-2-(4-sulfophenyl)-H-tetrazolium; IC_50_: half-maximal Inhibitory Concentration; HepG2: Hepatocarcinoma HL-60: promyelocytic leukaemia; SMMC-7721: human hepatocellular carcinoma; SW480: Human colon carcinoma; U-937: Histiocytic lymphoma.

**Table 4:** The antimicrobial activity of the extracts derived from *Ageratina* Spach.

Specie	PP/ES	Method	Microorganism	Result	Ref.
*A. adenophora*	AP/Methanol	μd	MRSA	MIC (mg/mL) 125	[20]
			*S. aureus*	25	
	AP/EO	μd	*E. coli*	MIC (μg/mL) >4000	[33]
			*P. aeruginosa*	>4000	
			*E. faecium*	>4000	
			*E. faecalis*	>4000	
			*S. aureus*	>4000	
	AP/EO	μd	*S. enterica* subsp. *enterica* serovar *Thypimurium*	>4000	
*A. janni*	L/EO	Df disc	*S. aureus*	MIC (mg/mL) 49.5	[40]
			*E. faecalis*	49.5	
*A.* *pentlandiana*	L/EO	μd	*S. aureus*	MIC (μL/mL) 11.9 + 0.1	[45]
			*B. subtilis*	22.7 ± 0.3	
			*E. coli*	57.7 ± 0.1	
			*S. thyphimurium*	41.6 ± 0.1	
		MBC	*S. aureus*	11.9 + 0.1	
			*B. subtilis*	22.7 ± 0.1	
			*E. coli*	64.8 ± 0.3	
			*S. thyphimurium*	50.0 ± 0.2	
*A. pichinchensis*	L/EO	Df disc	*S. aureus*	MIC (mg/mL) 104	[40]
			*E. faecalis*	104	
*A.* *tinifolia*	AP/EO	μd	*E. cloacae* clinical sample	>5	[46]
			*E. cloacae* ATCC	>5	
*A. havanensis*	L/Ethanol	SCA	HSV-1	EC_50_ (μg/mL) 809.9 ± 59.6	[37]
			HSV-2	1050 ± 42.9	
	L/EtOAc		HSV-1	311.6 ± 10.1	
			HSV-2	>450	
	L/n-butanol		HSV-1	>225	
			HSV-2	128.1 ± 22.8	
	S/Ethanol		HSV-1	>450	
			HSV-2	2614 ± 158	
	S/EtOAc		HSV-1	>450	
			HSV-2	>450	
	S/n-butanol		HSV-1	240.9 ± 5.7	
			HSV-2	145.4 ± 22.1	
	L&F/EtOAc	VIICE	HSV-1	463.4 ± 12.5	[38]
			HSV-2	>200	
*A. adenophora*	L/Ethanol	Df	*T. mentagrophytes*	MIC (mg/mL) 0.04	[19]
			*T. rubrum*	<0.0025	
*A. dendroides*	AP/EO	μd	*A. niger*	MIC (μg/mL) >4000	[33]
	AP/EO	μd	*C. albicans*	>4000	
*A. pichinchensis*	AP/(7:3) H-EtOAc	VS	*C. albicans*	Decrease % 81.2	[43]
*A. vacciniaefolia*	L/Ethanol	MTT	*T. cruzi*	EC_50_ (μg/mL) 256	[47]

L = leaves; AP = Aerial parts; S = Steams; H-EtOAc = Hexane-Ethyl acetate; EtOAc = Ethyl acetate; EO = Essential Oil, μd = microdilution; Df = diffusion; Df disc = diffusion disc; SCA = Suspension cell assay; VIICE = Virus inhibition induced cytopathic effect; VS = Vaginal suppositories (*in vivo* assay, patients with vulvovaginitis associated with *C. albicans* applied vaginally one suppository diary for 6 days); MIC: Minimal inhibitory concentration; MBC: Minimal bactericide concentration; ATCC: American Type Culture Collection; EC_50_: 50% Effective Concentration; Microorganisms: *Aspergillus niger*, *Bacillus subtilis*, *Candida albicans*, *Enterobacter cloacae, Enterococcus faecalis*, *Enterococcus faecium*, *Escherichia coli*, *Herpes simplex virus* type 1 (HSV-1) and type 2 (HSV-2), *Pseudomonas aeruginosa*, *Salmonella enterica* subsp. *enterica* serovar, *Salmonella thyphimurium*, *Staphylococcus aureus*, Methicillin-resistant *Staphylococcus aureus* (MRSA), *Tinea pedis*, *Trichophyton mentagrophytes*, *Trichophyton rubrum*, *Trypanosome cruzi*.

**Table 5:** The antimicrobial activity of the compounds obtained from *Ageratina* Spach.

Specie	Compounds	Method	Microorganism Assay	Result MIC (µg/mL)	Ref.
*A. adenophora*	2α-methoxyl-3β-methyl-6-(acetyl-*O*-methyl)-2,3-dihydrobenzofuran (**13**)	μd	*S. aureus*	25	[28]
			*B. cereus*	50	
			*B. subtilis*	25	
			*E. coli*	>100	
	1,6-dihydroxy-1-isopropyl-4,7-dimethyl-3,4-dihydronaphthalen-2(1*H*)-one (**14**)		*S. aureus*	>100	
			*B. cereus*	>100	
			*B. subtilis*	>100	
			*E. coli*	>100	
	Eupatorenone (**15**)		*S. aureus*	12.5	
			*B. cereus*	25	
			*B. subtilis*	25	
			*E. coli*	>100	
	3-hydroxymuurola-4,7(11)-dien-8-one (**16**)		*S. aureus*	25	
			*B. cereus*	12.5	
			*B. subtilis*	25	
			*E. coli*	>100	
	9-oxoageraphorone (**8**)		*S. aureus*	>100	
			*B. cereus*	>100	
			*B. subtilis*	>100	
			*E. coli*	>100	
	(4*R*,5*S*)-4-hydroxy-5-isopropyl-2-methyl-2-cyclohexehone (**17**)		*S. aureus*	>100	
			*B. cereus*	>100	
			*B. subtilis*	>100	
			*E. coli*	>100	
	7,9-diisobutyryloxy-8-ethoxythymol (**5**)	μd	*S. aureus*	>200	[27]
			*B. thuringiensis*	>200	
			*B. subtilis*	125	
			*E. coli*	>200	
			*S. dysenteriae*	>200	
	7-acetoxy-8-methoxy-9-isobutyryloxythymol (**6**)		*S. aureus*	125	
			*B. thuringiensis*	62.5	
			*B. subtilis*	62.5	
			*E. coli*	>200	
			*S. dysenteriae*	>200	
	7,9-di-isobutyryloxy-8-methoxythymol (**7**)		*S. aureus*	>200	
			*B. thuringiensis*	125	
			*B. subtilis*	125	
			*E. coli*	>200	
			*E. dysenteriae*	>200	
	9-oxoageraphorone (**8**)		*S. aureus*	>200	
			*B. thuringiensis*	>200	
			*B. subtilis*	>200	
			*E. coli*	>200	
			*S. dysenteriae*	>200	
	(−)-isochaminic acid (**9**)		*S. aureus*	31.3	
			*B. thuringiensis*	31.3	
			*B. subtilis*	15.6	
			*E. coli*	62.5	
			*S. dysenteriae*	62.5	
	(1α,6α)-10-hydroxy-3-carene-2-one (**10**)		*S. aureus*	15.6	
			*B. thuringiensis*	31.3	
			*B. subtilis*	15.6	
			*E. coli*	62.5	
			*S. dysenteriae*	62.5	
	5-*O*-trans-*o*-coumaroylquinic acid methyl ester (**18**)	μd	*S. aureus*	MIC (µM) 88.8	[24]
			*B. thuringiensis*	88.8	
			*E. coli*	88.8	
			*S. enterica*	88.8	
			*S. dysenteriae*	177.6	
	Chlorogenic acid methyl ester (**19**)		*S. aureus*	84.8	
			*B. thuringiensis*	84.8	
			*E. coli*	84.8	
			*S. enterica*	84.8	
			*S. dysenteriae*	169.8	
	Macranthoin F (**20**)		*S. aureus*	29.4	
			*B. thuringiensis*	59.0	
			*E. coli*	59.0	
			*S. enterica*	14.7	
			*S. dysenteriae*	117.9	
	Macranthoin G (**21**)		*S. aureus*	59.0	
			*B. thuringiensis*	59.0	
			*E. coli*	59.0	
			*S. enterica*	7.4	
			*S. dysenteriae*	117.9	
*A. deltoidea*	Grandiflorenic acid (**22**)	μd	*S. aureus*	MIC (µg/mL) 31	[32]
	Kaurenoic acid (**23**)		*S. aureus*	31	
	Deltoidin A (**24**)		*E. coli*	16	
	8β-angeloyloxyelemacronquistianthus acid (**25**)		*S. aureus*	125	
			*E. coli*	125	
*A. glabrata*	(8*S*)-10-Benzoyloxy-8,9-epoxy-6-hydroxythymol isobutyrate (**26)**	MTT/PMS	*E. histolytica*	IC_50_ (µM) 1.6	[35]
			*G. lamblia*	36.9	
	10-Benzoyloxy-8,9-epoxy-6-acetyloxythymol isobutyrate (**27**)		*E. histolytica*	0.84	
			*G. lamblia*	24.2	
	10-Benzoyloxy-8,9-epoxy-6-methoxythymol isobutyrate (**28**)		*E. histolytica*	169.6	
			*G. lamblia*	191.2	
	10-Benzoyloxy-8,9-epoxythymol isobutyrate (**29**)		*E. histolytica*	25.9	
			*G. lamblia*	48.3	
	10-Benzoyloxy-8,9-dehydro-6-hydroxythymol isobutyrate (**30**)		*E. histolytica*	61.2	
			*G. lamblia*	68.0	
	10-Benzoyloxy-6,8-dihydroxy-9-isobutyryloxythymol (**31**)		*E. histolytica*	45.6	
			*G. lamblia*	60.7	
	Pectolinaringenin (**32**)		*E. histolytica*	43.6	
			*G. lamblia*	68.7	
	(8*S*)-8,9-epoxy-6-hydroxy-10-benzoyloxy-7-oxothymol isobutyrate (**33**)		*E. histolytica*	184.9	
			*G. lamblia*	167.4	
*A. jocotepecana*	(−)-(5*S*,9*S*,10*S*,13*S*)-labd-7-en-15-oic acid (**34**)	μd	*S. aureus*	MIC (mg/mL) 0.78	[41]
			*B. subtilis*	0.15	
	(−)-(5*S*,9*S*,10*S*,13*Z*)-labda-7,13-dien-15-oic acid (**35**)		*S. aureus*	1.00	
			*B. subtilis*	10.00	
	(+)-(5*S*,8*R*,9*R*,10*S*,13*R*)-8-hydroxylabdan-15-oic acid (**36**)		*S. aureus*	2.34	
			*B. subtilis*	1.56	
*A. adenophora*	Encecalin (**37**)	Df disc (50 μg/disc)	*F. oxysporum* f. sp. *niveum*	DIZ (mm) 10.00 ± 0.15	[25]
	7-hydroxy-dehydrotremetone (**38**)		*C. gloeosporioides*	17.28 ± 0.46	
			*C. musae*	17.24 ± 0.52	
			*R. solani*	16.40 ± 0.81	
			*F. oxysporum* f. sp. *niveum*	13.90 ± 1.05	
			*A. alternata*	14.36 ± 0.68	
*A. cylindrica*	Cylindrinol B (**39**)	NA	*E. histolytica*	IC_50_ (µM) 287.5	[31]
			*G. lamblia*	226.2	
	Cylindrinol C (**40**)		*E. histolytica*	237.0	
			*G. lamblia*	251.9	
	Cylindrinol D (**41**)		*E. histolytica*	86.9	
			*G. lamblia*	134.0	
	Cylindrinol E (**42**)		*E. histolytica*	210.2	
			*G. lamblia*	164.5	
	Cylindrinol F (**43**)		*E. histolytica*	213.2	
			*G. lamblia*	151.1	
	ent-15β-(β-L-fucosyloxy)kaur-16-en-19-oic acid β-Dglucopyranosyl ester (**44**)		*E. histolytica*	43.3	[30]
			*G. lamblia*	41.9	
	ent-15β-(4-acetoxy-β-L-fucosyloxy) kaur-16-en-19-oic acid β-D-glucopyranosyl ester (**45**)		*E. histolytica*	49.5	
			*G. lamblia*	69.5	
	ent-15β-(3-acetoxy-β-L-fucosyloxy) kaur-16-en-19-oic acid β-D-glucopyranosyl ester (**46**)		*E. histolytica*	52.7	
			*G. lamblia*	48.9	
	ent-15β-(β-L-fucosyloxy)kaur-16-en-19-oic acid (**47**)		*E. histolytica*	73.5	
			*G. lamblia*	98.5	
	(8*S*)-8,9-epoxy-6-hydroxy-10-benzoyloxy-7-oxothymol isobutyrate (**33**)		*E. histolytica*	184.9	[29]
			*G. lamblia*	167.4	

MIC = Minimal inhibitory concentration; μd = microdilution; Df disc = Diffusion disc; DIZ = Diameter of Inhibitory Zone; MTT/PMS = Tetrazolium salt/phenazine methosulfate; IC_50_ = Fifth inhibitory concentration; NA = Nitazoxanide assay; Microorganisms: *Alternaria alternata*; *Bacillus cereus*; *Bacillus subtilis*; *Bacillus thuringiensis*; *Colletotrichum gloeosporioides*; *Colletotrichum musae*; *Entamoeba histolytica*; *Escherichia coli; Fusarium oxysporum* f. sp. *niveum*; *Giardia lamblia*; *Salmonella enterica*; *Shigella dysenteriae*; *Staphylococcus aureus*; *Rhizoctonia solani*.

## Data Availability

Data sharing is not applicable to this article as no new datasets were created or analysed in this study. All data used were obtained from publicly available literature accessed via the Springer^®^, PubMed^®^, ScienceDirect^®^, and Google Scholar^®^ online databases, as described in the Methods Section.

## Abbreviations

The following abbreviations are used in this manuscript:

A549	Lung cancer
AP	Aerial parts
ATTC	American Type Culture Collection
BioMod	Biological model
Bax	Bcl-2-associated X protein
Bcl-2	B-cell lymphoma 2
CCL	Cancer cell line
CC_50_	50% Cytotoxic Concentration
Caco-2	Colon cancer
Calu-1	Epidermoid carcinoma of the lung
DNA	Deoxyribonucleic acid
DCM	Dichloromethane
Df	Diffusion
Df disc	Diffusion disc
DIZ	Diameter of Inhibitory Zone
EC_50_	50% Effective Concentration
EtOAc	Ethyl acetate
EO-Hdest	Essential oil by Hydro distillation
EO	Essential Oil
F	Flowers
FS	Flowering stage
IC_50_	Fifth inhibitory concentration
H-EtOAc	Hexane-Ethyl acetate
HCT-116	Adherent cell of colon cancer
HCT-15	Colorectal carcinoma
HeLa	Cervical carcinoma
HepG2	Hepatocarcinoma
HT29	Colorectal adenocarcinoma
HL-60	Promyelocytic leukaemia
HSV-1	*Herpes simplex virus* type 1
HSV-2	*Herpes simplex virus* type 1
I	Inflorescence
IC_50_	Half-maximal Inhibitory Concentration
KB	Epithelial carcinoma
L	Leaves
LC_50_	Fifth Lethal Concentration;
L&F	Leaves and flowers
L&S	Leaves and stems
m	Meters
MIC	Minimal inhibitory concentration
MBC	Minimal bactericide concentration
MDA-MB-231	Human breast cancer
MCF-7	Breast cancer
MRSA	Methicillin-resistant *Staphylococcus aureus*
MTT	3-[4,5-dimethylthiazol-2-yl]-2,5 diphenyl tetrazolium bromide
MTS	3-(4,5-dimethylthiazol-2-yl)-5-(3-carboxymethoxyphenyl)-2-(4-sulfophenyl)-*H*-tetrazolium
MTT/PMS	Tetrazolium salt/phenazine methosulfate
μd	microdilution
NA	Nitazoxanide assay
NK-kB	Nuclear Factor kappa-light-chain-enhancer of activated B cells
OVCAR	Ovarian adenocarcinoma
PC-3	Prostatic adenocarcinoma
PP/ES	Part of the plant/extraction solvent
R	Root(s)
S	Stems
SiHa	Uterine squamous cell carcinoma
SCA	Suspension cell assay
SMMC-7721	uman hepatocellular carcinoma
SW480	Human colon carcinoma
4T1	Breast cancer
U-937	Histiocytic lymphoma
UISO	Merkel carcinoma cell
VIICE	Virus inhibition induced cytopathic effect
VS	Vaginal suppositories
VSt	Vegetative stage
Vero	kidney-derived epithelial cell
WHO	World Health Organization
